# Hemodynamic, metabolic and inflammatory effects of dexmedetomidine in a pig model of septic shock

**DOI:** 10.1186/cc9774

**Published:** 2011-03-11

**Authors:** P Carnicelli, D Fantoni, D Otsuki, A Monteiro Filho, M Kahvegian, J Noel-Morgan, J Auler

**Affiliations:** 1 Faculdade de Medicina Veterinária e Zootecnia da Universidade de São Paulo, Brazil; 2 Faculdade de Medicina da Universidade de São Paulo, Brazil

## Introduction

The use of dexmedetomidine to achieve sedation, analgesia and mechanical ventilation has increased in critically ill patients, although little is known about its effects in septic shock. The aim of this study was to assess hemodynamic, metabolic and inflammatory effects of dexmedetomidine in a pig model of septic shock.

## Methods

Eighteen pigs were anesthetized, mechanically ventilated and randomly allocated into three groups of six animals: sham group, shock group (intravenous infusion of live *Escherichia coli *over 1 hour) and shock+dex group (*E. coli *+ bolus and constant rate infusion treatment with dexmedetomidine). Both shock groups received fluid therapy with lactated Ringer's (LR) and norepinephrine to reach central venous pressure of 8 to 12 mmHg and mean arterial pressure ≥65 mmHg. T0 was considered the end of bacterial infusion and animals were monitored hourly for 240 minutes. Hemodynamic parameters were assessed with a pulmonary artery catheter and femoral arterial catheter. Blood gases, intestinal tonometry and inflammatory cytokines were also measured. Two-way ANOVA and Tukey test were used for statistical analysis (*P *< 0.05).

## Results

*E. coli *infusion resulted in cardiovascular collapse, acute lung injury and metabolic acidosis. At T0, oxygen consumption was significantly greater in the shock+dex group (149.9 ± 25.6 ml/minute/m^2^) than in the shock group (111.5 ± 21.6 ml/minute/m^2^), as was Pr-Pa (53 ± 14 mmHg and 35 ± 11 mmHg, respectively). At T180, SvO_2 _in the shock+dex group was statistically lower than in the shock group (62.5 ± 9.0 vs. 74.2 ± 9.1%, respectively). At T240, cardiac index in the shock+dex group was lower than that in the shock and sham groups (2.8 ± 0.5 vs. 3.6 ± 1.7 vs. 4.7 ± 1.1 ml/minute/m^2^, respectively) while the oxygen extraction rate was larger in the shock+dex group (43 ± 20%) than in the shock group (25 ± 11%). TNFα levels were similar in both groups. Although plasma levels of IL-1β, IL-6 and IL-10 were elevated in the shock group, there was no statistical significance with the shock+dex group. No statistical difference was found in treatment with LR or norepinephrine, nor in urine output.

## Conclusions

Dexmedetomidine is likely to cause a mismatch between oxygen delivery and consumption by affecting microcirculation in critically ill patients, despite treatment with crystalloids and vasoactive agents. Its effects on the inflammatory response remain unclear.

